# Thermal Imaging as a Method to Indirectly Assess Peripheral Vascular Integrity and Tissue Viability in Veterinary Medicine: Animal Models and Clinical Applications

**DOI:** 10.3390/ani14010142

**Published:** 2023-12-31

**Authors:** Daniel Mota-Rojas, Asahi Ogi, Dina Villanueva-García, Ismael Hernández-Ávalos, Alejandro Casas-Alvarado, Adriana Domínguez-Oliva, Pamela Lendez, Marcelo Ghezzi

**Affiliations:** 1 Neurophysiology of Pain, Behavior and Assessment of Welfare in Domestic Animals, DPAA, Universidad Autónoma Metropolitana (UAM), Mexico City 14389, Mexico; 2 Department of Neurobiology and Molecular Medicine, IRCCS Fondazione Stella Maris, 56128 Pisa, Italy; 3 Division of Neonatology, Hospital Infantil de México Federico Gómez, Mexico City 06720, Mexico; 4 Clinical Pharmacology and Veterinary Anesthesia, Biological Sciences Department, FESC, Universidad Nacional Autónoma de México, Cuautitlán 54714, Mexico; 5 Anatomy Area, Faculty of Veterinary Sciences (FCV), Universidad Nacional del Centro de la Provincia de Buenos Aires (UNCPBA), University Campus, Tandil 7000, Argentina; 6 Animal Welfare Area, Faculty of Veterinary Sciences (FCV), Universidad Nacional del Centro de la Provincia de Buenos Aires (UNCPBA), University Campus, Tandil 7000, Argentina

**Keywords:** thrombosis, ischemia, burn wounds, skin grafting, free flaps, wound healing

## Abstract

**Simple Summary:**

Infrared thermography can indirectly assess peripheral vascular diseases because skin surface temperature depends on blood flow and heat dissipation through skin microvasculature. The present review aims to summarize and analyze the application of infrared thermography in veterinary medicine as a method to indirectly assess peripheral vascular integrity and its relation to the amount of radiated heat and as a diagnostic technique for tissue viability, degree of damage, and wound care. Although additional research is needed to establish the utility of thermal imaging to monitor vascular integrity in veterinary medicine, studies performed in animals diagnosed with thromboembolisms or thermal impairment due to neuropathies or are in need of burn wound management suggest a promising application of infrared thermography to evaluate peripheral vascular alterations and tissue viability.

**Abstract:**

Infrared thermography (IRT) is a technique that indirectly assesses peripheral blood circulation and its resulting amount of radiated heat. Due to these properties, thermal imaging is currently applied in human medicine to noninvasively evaluate peripheral vascular disorders such as thrombosis, thromboembolisms, and other ischemic processes. Moreover, tissular damage (e.g., burn injuries) also causes microvasculature compromise. Therefore, thermography can be applied to determine the degree of damage according to the viability of tissues and blood vessels, and it can also be used as a technique to monitor skin transplant procedures such as grafting and free flaps. The present review aims to summarize and analyze the application of IRT in veterinary medicine as a method to indirectly assess peripheral vascular integrity and its relation to the amount of radiated heat and as a diagnostic technique for tissue viability, degree of damage, and wound care.

## 1. Introduction

Infrared thermography (IRT) is widely used in human and animal medicine as a noninvasive and complementary diagnostic tool for the early detection of inflammatory processes [1,2,3,4] and peripheral vascular disorders where blood flow is compromised [5,6]. This is due to its potential to indirectly evaluate the volume of blood flow to the skin [7]. Therefore, any disturbance to normal blood circulation can potentially alter the amount of radiated heat from the skin of mammals [5]. Examples of this in humans include thrombosis, thromboembolisms, vasculitis, vascular injuries, lupus, diabetic foot, and ischemia processes where peripheral blood supply is abnormal [6,8,9,10].

Contrary to human medicine, the application of IRT to assess vascular compromise in veterinary medicine is still developing, and limited studies have been published. However, it has been suggested that identifying an altered local temperature response in animals might help to recognize blood perfusion disorders such as vasculitis [11] or even discern between benign and malign neoplasia [12,13]. For example, aortic thromboembolism cases in cats show a hypothermic response in pelvic limbs, a method that can serve to differentiate between healthy animals and those affected by the pathology [14]. Another example is burn injuries, for which determining the degree of damage is essential to adopt treatment protocols and estimate recovery time [15,16,17]. 

Canine, swine, and murine models show that decreases in temperature in the burn wound site are associated with a higher burn degree (e.g., full-thickness) [18] and that this is also related to a poor prognosis when skin-grafting or free-flap surgeries are performed, due to vascular alterations [19,20,21]. Likewise, indocyanine green imaging and IRT were used to evaluate tissue blood flow in a porcine model of intestinal resection, and the authors found that IRT can differentiate well-blooded intestinal segments [22]. Moreover, IRT has been applied to determine skin transplant viability and wound healing progress [17,23,24].

Therefore, due to the wide field of research in which IRT can be used to identify medical processes where blood flow is altered, the present review aims to summarize and analyze the application of IRT to evaluate peripheral vascular disorders and blood flow integrity in skin transplant procedures in veterinary medicine, in both a clinical and an experimental setting. 

## 2. Search Methodology

The literature search was performed using the Web of Science and PubMed databases. Major keywords used for the search were the combination of “infrared thermography” or “thermal imaging” with “peripheral vascular disorder animal models”, “animal peripheral ischemic processes”, “skin grafting in animals”, and “animal models of burn injuries”. The selected papers included studies where IRT—alone or together with other techniques—was used to evaluate vascular integrity or thermal changes in clinical cases or experimental models of peripheral ischemia. The search also considered human studies to compare with and use as a base, since current research regarding vascular disorders and veterinary medicine is limited. Due to this, the search included a range of different animal species. 

## 3. Heat Radiation and Its Relationship with Tissular Perfusion

All objects with a temperature above absolute zero are known to emit thermal radiation [6,25]. IRT detects this infrared radiation, and the average emissivity of the skin (0.98) makes it a material appropriate for thermal imaging [26,27,28,29,30]. Therefore, distinctive thermal changes and local changes in heat production can be detected due to an affected blood flow pattern or certain pathologies [31]. 

**Figure 1:**
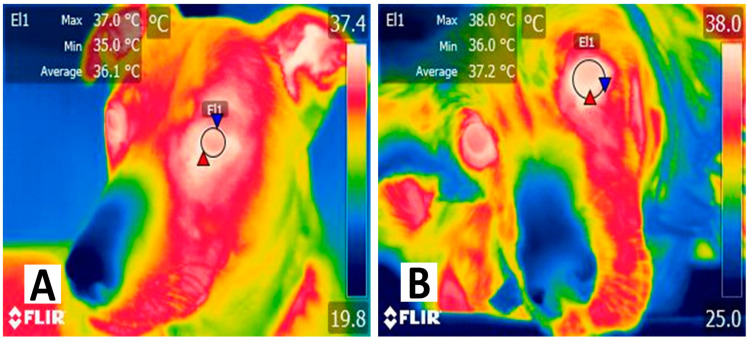
The surface temperature of a dog undergoing an infectious disease. (**A**) Thermal response in a healthy 3-year-old mixed female dog. The maximum (red triangle) surface temperature of the periocular region (El1) shows a value of 37 °C, while the minimum (blue triangle) and average temperatures were 36 °C and 35 °C, respectively. (**B**) Thermal response in a 7-year-old female English Setter dog diagnosed with pyometra. It is observed that the surface temperature of the periocular region (El1) is 1 °C higher than the maximum (red triangle) and average temperature of a healthy dog. Regarding the minimum temperature (blue triangle), a difference of +1.1 °C was found when compared to a healthy animal. The presence of cytokines, such as interleukin (IL)-1, IL-6, and prostaglandin F2 alpha, induces fever. The increase in body core temperature is reflected in the vasodilation of superficial blood vessels to increase heat dissipation. Therefore, IRT can help recognize febrile states in animals. Radiometric images were obtained using a T1020 FLIR thermal camera. Image resolution 1024 × 768; up to 3.1 MP with UltraMax. FLIR Systems, Inc. Wilsonville, OR, USA.

To understand how IRT is related to heat production and tissue perfusion, it is necessary to address how circulation can affect surface heat radiation. According to Morrison and Nakamura [32], peripheral thermoreceptors in the skin detect thermal changes. Transient receptor potential (TRP) ion channels are the main thermoreceptors able to detect a wide range of temperatures from heat/cold to noxious heat/cold [33]. In particular, TRP of the vanilloid family (TRPV) can respond to both thermal (noxious/painful heat) and nonthermal inputs such as the release of chemical mediators [e.g., histamine, serotonin, interleukin (IL)-6, IL-10, and tumor necrosis factor-alpha (TNF–α)], elements present during the inflammatory process and tissular injury [34,35], triggering nocifensive behaviors (skin vasomotor responses or behavioral changes) to restore thermostability and increase or decrease heat loss [36,37,38]. After stimuli detection, hypothalamic centers such as the preoptic area, the anterior hypothalamus, and the dorsomedial hypothalamus are activated to process thermal information received from the lateral parabrachial nucleus of the pons [39,40]. For example, Figure 1 shows the facial thermal response of a febrile canine patient due to an infectious process. When comparing the temperature of the febrile animal with a healthy patient, a difference of up to 1.1 °C can be observed in the periocular region. Diego et al. [41] reported a similar pattern in febrile ovine experimentally infected with bluetongue virus serotypes 1 and 8. The authors found that ocular infrared temperature was moderately positively correlated with the rectal temperature (r = 0.504) and that IRT could discriminate between febrile and nonfebrile animals with a sensitivity of 85% and a specificity of 97%. 

Apart from general changes in temperature, circulatory disorders in skin blood vessels can change heat distribution and, therefore, variations in heat emissions through the skin [21]. Finstad [31] reported this in 49 client-owned dogs undergoing laparotomy to correct foreign body obstructions, a pathology known to compress intestinal blood vessels and cause tissular necrosis if not treated correctly. In the study, IRT was assessed using an infrared camera held 1 m from the obstructed small intestine portion. The average surface temperature of the small intestine directly over the foreign body was 2.4 °C lower than the rest of the intestine (healthy tissue), particularly when it was a hard material (3.2 to 5.2 °C). Although these findings did not show a correlation with lactate or blood pressure, IRT was able to detect vascular compromise due to the foreign body. 

**Figure 2:**
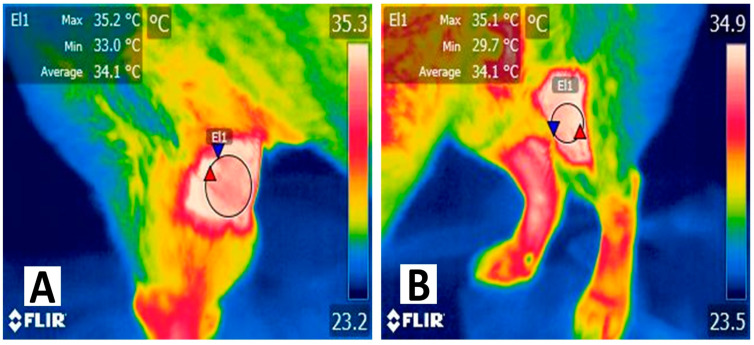
Thermal response associated with an inflammatory process in a dog with a knee fracture. (**A**) Thermal response in a 6-year-old female Shiba Inu dog with inflammation. Temperature differences can be observed in the right pelvic limb with a fracture in the femoral–tibial–patellar joint. The maximum surface temperature (red triangle) of the region (El1) was 35.2 °C, with an average temperature of 34.1 °C and a minimum of 33 °C (blue triangle). (**B**) Thermal response in a healthy knee. In the same animal, the thermal response of the femoral–tibial–patellar joint (El1) of the left hindlimb had a difference of up to 3.3 °C when comparing the average surface temperature of both knees. The release of pro-inflammatory mediators (e.g., IL-1, IL-6, IL-10, prostaglandins, serotonin, and histamine) produces vasodilation of capillaries in the dermal tissue, increasing heat dissipation and radiation.

Similarly, during other inflammatory processes, as shown in Figure 2 in the knee joint, differences in temperature in the affected limb can be observed when compared to the healthy side. In these cases, and during burn injuries, the inflammatory process is the result of the release of pro-inflammatory mediators (e.g., prostaglandin F2 alpha, histamine, serotonin, IL-6, IL-10, and TNFα), substances that produce vasodilation of the superficial blood capillaries and increase heat radiation through the skin [42].

**Figure 3:**
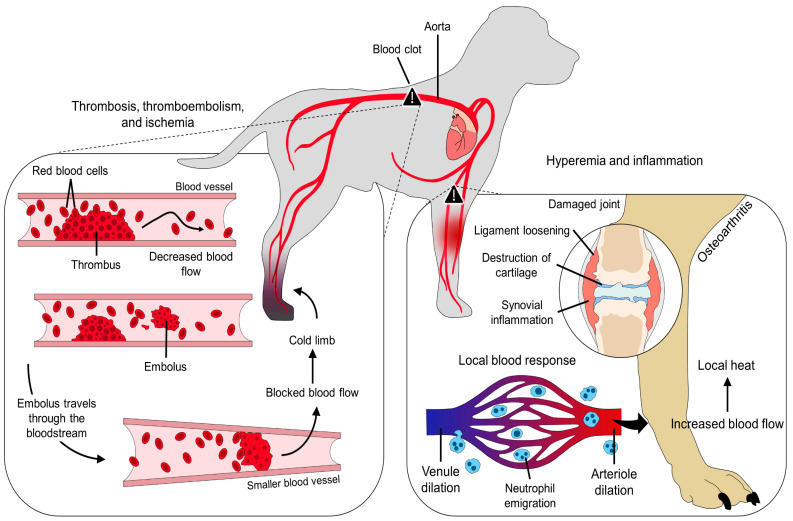
Peripheral vascular changes due to ischemic and inflammatory processes. Whether a decreased or increased blood flow is present, changes in the local temperature arise due to alterations in the normal blood flow. For example, during thromboembolisms, initially, the thrombus decreases blood flow by obstructing part of the blood vessel. However, the vessel can be completely blocked when an embolus travels through the bloodstream. Locally, this will stop or significantly reduce blood flow, decreasing the local surface temperature. On the contrary, during an inflammatory process such as osteoarthritis, vasodilation of venules and arterioles increases blood flow to promote neutrophil emigration. The release of inflammatory mediators also causes vasodilation, increasing local heat radiation.

Therefore, peripheral hemodynamic changes due to a general condition (e.g., fever) or to a local injury (e.g., inflammatory process or burn wound) will undoubtedly modify skin surface temperature, a parameter that is influenced by critical factors such as local blood circulation, heat conduction by deeper tissues, and heat loss [26]. Figure 3 schematizes the vascular changes observed during the ischemic and inflammatory processes. 

In large animals such as ruminants, early detection of inflammatory processes such as laminitis could help to prevent complications in the hooves. In this sense, IRT could be applied as a complementary tool to traditional diagnostic methods, as reported by Lin et al. [43], who found an optimal threshold based on actual foot temperature of 23.3 °C to detect healthy limbs from those affected by laminitis, with a sensibility of 78.5% and specificity of 39.2%. Due to these properties, thermographic imaging could be a reliable and real-time clinical tool to detect inflammatory-related events or abnormal blood flow processes.

## 4. Peripheral Vascular Disorders and Thermography

The association between the control of surface temperature and the vasomotor response suggests that any vascular involvement disorders can alter the thermal response of tissues [44]. An example of this is a diabetic foot, a pathology where vascular compromise is present, as mentioned by Ilo et al. [24], who compared the thermal response of human patients with suspected diabetic feet (n = 118) and healthy patients (n = 93). The surface temperature of the dorsal and plantar regions of the foot was significantly higher (33% higher) in patients with diabetic foot by 2 °C. Moreover, thermal findings coincided with decreases in pressure in the toes to 50 mmHg. A similar influence of blood pressure in limb surface temperature was reported in piglets, in whom a negative correlation was found between temperature and blood pressure [45]. These results suggest that the neuropathies occurring in diabetes patients generate microcirculatory alterations that have consequences on the thermoregulatory mechanisms of peripheral regions such as the foot. Furthermore, diabetes is also linked to arteriovenous shunting [46]. These arteriovenous anastomoses are innervated by sympathetic vasoconstrictor nerves that allow high flow rates in low-resistance vessels. States of local capillary hypoperfusion can lead to impairments in wound healing [47,48]. Thus, local and systemic disorders can alter peripheral blood flow and, in the case of hypoperfusion, these changes can be observed with IRT as a decrease in heat elimination.

**Figure 4:**
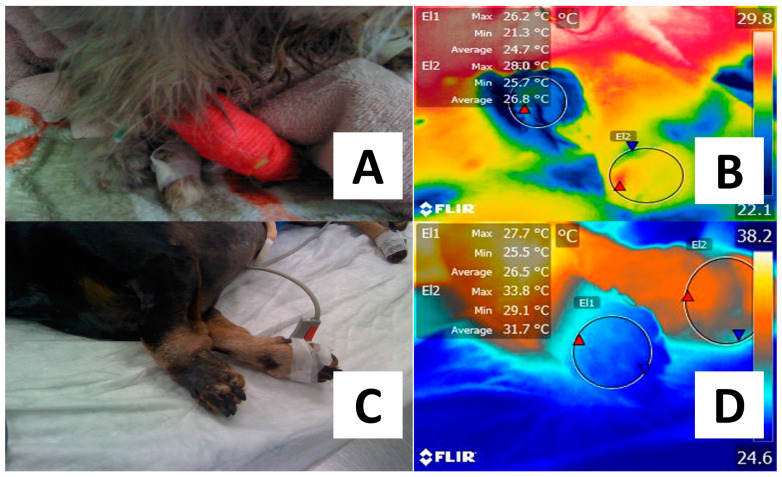
Difference in the thermal response of patients diagnosed with peripheral vascular alterations. (**A**) Persian male cat diagnosed with aortic thromboembolism after evaluating clinical signs such as pain on palpation in the right pelvic limb, absence of pulse, cold limb, and mobility difficulty. (**B**) The phalangeal region of the affected right hindlimb (El1) showed lower temperatures of up to 3.3 °C when compared to the healthy left hindlimb (El2). (**C**) A four-year-old male Dachshund dog diagnosed with thrombosis due to a secondary liver infection was presented with a reduced perfusion in the left forelimb. Necrosis can be observed. (**D**) With thermal imaging, it can be observed that the average surface temperature of the phalangeal region of the right forelimb (El2) is 5.2 °C higher than the same region in the affected limb (El1). The explanation for this thermal response is that the presence of a thrombus obstructs blood flow due to occlusion at the arterial level. Thus, the decrease in blood flow has an impact on local heat response. Maximal temperature is indicated with a red triangle and the minimal with a blue triangle. Radiometric images were obtained using a T1020 FLIR thermal camera. Image resolution 1024 × 768; up to 3.1 MP with UltraMax. FLIR Systems, Inc. Wilsonville, OR, USA.

Another pathology where IRT has been suggested to diagnose and monitor vascular alterations is arterial thromboembolism. A study carried out by Pouzot-Nevoret et al. [14] evaluated the application of IRT to differentiate between nonischemic and aortic thromboembolism in 16 cats (healthy cats = 10 and sick cats = 6). By assessing the temperature of the pelvic limbs, the authors found that in animals with aortic thromboembolism, limb temperature was significantly lower (by 3.3 °C) than the values recorded in healthy cats. Furthermore, a decrease of 2.4 °C was set as a reference to differentiate healthy from affected animals, with a sensitivity of 80% and a specificity of 100%. Since a thrombus obstructs normal blood circulation, the decrease in surface temperature is an expected response that can be detected through IRT [44]. Figure 4 schematizes a feline patient diagnosed with aortic thromboembolism and the thermal response evaluated through IRT.

Postmortem evaluations using IRT to detect thermal patterns during coronary heart disease have been studied in pigs. Thermal imaging coronarography enabled the evaluation of coronary vessels in ex situ swine hearts [49]. Although this was a postmortem evaluation, further analysis is required to apply this technique in veterinary medicine. Experimental hypoperfusion studies in rats also used IRT to visualize vasodilation of choked vessels in a model of delayed pedicled perforator flap rat model [50]. In 43 rats divided according to the experimental treatment, after the use of a flap and after strangulation of the thoracodorsal artery, a red zone was associated with a 1.4% increase in local temperature and a 0.3 mm increase in diameter of the same artery compared to the iliac artery [50]. According to the authors, this result is consistent with the changes due to the congestion process that occurs after the obstruction. The same occurs when a thrombus is present, leading to hypoperfusion and a subsequent decrease in local temperature. IRT was used in the same species to detect tissue perfusion disorders in rats [51]. In animals with unilateral or bilateral femoral vessel ligation, statistically significant differences in thigh, shank, and foot temperature were observed. A slow return in the temperature of the shank and foot was observed in the ligated limbs when compared with healthy limbs [51]. 

Similarly, a study aimed to evaluate variations in surface temperature after rapid hemodynamic changes in eight healthy piglets under general anesthesia. The animals were exposed to different blood pressure levels and monitored through IRT. The surface temperature of the left forelimb had a negative correlation with the temperature gradient and blood pressure (r = −0.042), which led the authors to conclude that IRT is a tool for detecting early changes in peripheral perfusion [45]. In this sense, Caramalac et al. [52] evaluated a 9-year-old dog with clinical signs of a cold limb without a pulse, proprioception, and deep pain. Due to the clinical signs, thromboembolism was a presumptive diagnosis. IRT was able to determine a difference of 3.7 °C between the affected limb and the healthy one.

Therefore, the scientific evidence suggests that IRT is a promising healthcare tool for the evaluation of vascular compromise in peripheral tissues. In addition, it could help to detect early changes before a chronic or severe stage can be developed. Table 1 summarizes the animal studies where vascular disorders were reported. 

## 5. Thermal Imaging Applied to Assess Tissular Damage Degree and Microvascular Repair

Because IRT can remotely evaluate the vascular integrity of tissues, thermal imaging has also been applied to monitor the degree of tissular damage and blood perfusion restoration [15,16,17]. Different studies in humans showed that blood flow assessment can predict the time of healing [15,53,54]. This is of clinical relevance in burn patients in whom IRT can help to assess burn depth (e.g., superficial, superficial partial-thickness, deep partial-, or full-thickness) and estimate the recovery time [16]. These studies show that IRT can help to categorize the deepness of the injury as a primary and real-time technology for burn wound management, including surgical repair using skin grafting or free flaps to monitor tissue viability and improve the healing process [55]. Although the common procedure to evaluate reconstructive surgery is by looking at granulation tissue and proper healing, the ability of IRT to detect vascular imbalances could help monitor the success of skin transplantation. Since skin transplants need an integrated microvascular blood flow to survive, vascular alterations are the main reason for failure. This is relevant because the failure rate of skin grafts and free flaps is around 2.5–23.7% and 0.5–23.8%, respectively [20].

In the case of burn patients, there are different types of burn injuries: first-degree burn or superficial wounds; second-degree or superficial partial-thickness burn; third-degree or deep partial-thickness wound; and fourth-degree or full-thickness injury [56]. According to the degree of damage, IRT can detect the zones where the integrity of vascular structures is maintained, differentiate between abnormal heat transfer of burn zones, and serve as a guide to performing grafts or flaps [19,53]. Identification of the degree and heat pattern changes is due to the different zones that are present in burns: (1) coagulation zone of irreversible necrosis; (2) stasis zone with damage of the vasculature; and (3) hyperemia zone of edematous nature and with tissue that can recover [53]. Prindeze et al. [57] mention that the hyperemia zone, using ADT in humans, can be observed as a zone with higher temperatures when compared to the coagulation and stasis zones. Moreover, after a burn injury, the permeability of capillaries and venules increases, causing edema and hypovolemia [58]. Additionally, the release of pro-inflammatory mediators such as histamine, bradykinin, cytokines, and platelet-activating factors alters vascular permeability [58]. 

The vascular damage and the altered heat transfer are physiological responses that can be monitored through IRT. Infrared evaluation applied to burn depth assessment in humans showed a sensitivity and specificity of 84% and 76%, respectively [16], while visual evaluation had an accuracy of 60% [59]. Moreover, the application of IRT could guide surgeons in deciding burn wound treatment, whether conservative or surgical [60]. 

In contrast to human medicine, burn wound management (including diagnosis and treatment) is challenging and uncommon in veterinary medicine [61]. In small animal medicine, most thermal burn cases are associated with accidental burns caused by supplemental heat (e.g., thermal mattresses) [62]. Some studies made in animal models have shown the application of IRT to burns, such as Lawson [18], who was the first researcher using thermal imaging to determine burn depth in dogs and measure cutaneous blood flow [63]. Porcine models of burn injury in Yorkshire pigs showed that a progressive increase and warmer surface temperatures (from 29.99 ± 0.63 °C to 30.93 ± 0.47 °C) during the first 7 days after burn injury was related to lower scar depth at 28 days after injury (1.29 ± 0.68 mm). Contrarily, pigs with surface hypothermia (from 31.00 ± 0.71 °C to 27.31 ± 0.37 °C) resulted in larger scar depth (4.68 ± 0.60 mm) [59]. These results suggest that the surface temperature of the injury during the first days can predict burn depth, and this is relevant because it is known that, in humans, first-degree and second-degree burns usually halt within three weeks, while third-degree ones require more than three weeks to heal and might develop hypertrophic scars [64]. Similarly, evaluation of the thermal time constant using active dynamic thermography (ADT) in pigs showed a high correlation between surface temperature and predicted healing time with an accuracy of 83.0%, close to the accuracy of histopathologic assessment (84.0%) [60]. The authors found that a shorter thermal time (49.1 ± 23 s) was associated with wound self-healing within three weeks, while a longer time (86.3 ± 32.3 s) resulted in an extended healing period [60]. 

In another swine model, high-resolution IRT was used to determine its usefulness in predicting burn severity during the first four days after injury [65]. In Yorkshire pigs, the surface temperature decreased with increasing burn severity, meaning that superficial burn wounds had the warmest temperatures (approximately 33.7 °C to 35 °C), while full-thickness wounds had progressive lower temperatures (approximately 32.4 °C to 33 °C). Additionally, IRT was able to predict histological changes such as necrosis, apoptosis, and vascular occlusion with correlation values between 0.62 and 0.13 [65]. The same pattern has been observed in human patients, in whom Hardwicke et al. [66] reported that when compared to nonburn patients, the skin temperatures of full-thickness and deep partial burns were significantly cooler (−2.3 °C and −1.2 °C, respectively), while superficial partial-thickness burns had no significant differences of 0.1 °C.

Between grafts and flaps, there are vascular differences that need to be considered when using IRT. On one side, grafts are a type of skin transplant (dermis, epidermis, or both) completely detached from the blood supply. Grafts need a well-vascularized bed wound to survive [67]. This means that the free segment of skin needs to reestablish a vascular supply to consider it a successful grafting procedure [68]. Grafts are put on a well-vascularized bed. In contrast, a flap has an integral blood supply [67]. 

Klama-Baryla et al.’s [21] study shows the application of IRT to evaluate the survivability of autologous split-thickness engraftment in patients suffering thermal injuries. Consecutive monitoring at days 0, 5, 14, and 21 showed that a progressive increase in surface temperature between 1.21 °C and 3.1 °C immediately after the graft indicated a normal healing process with angiogenesis (confirmed with angiography). Contrarily, graft loss occurred when low temperatures around 32.9 °C were observed. IRT can also help to predict treatment by either reepithelization, skin graft, or amputation [69,70]. This was determined in burn patients with partial- or full-thickness burns in extremities [69]. The difference of temperatures between measuring points (ΔT) showed an average ΔT of 1.77 ± 0.92 °C for superficial and deep partial-thickness wounds, while full-thickness burns had a ΔT of 5.45 ± 2.86 °C. Regarding the predictive value of IRT, ΔT was 1.75 ± 0.89 °C for the patients that healed by reepithelization. In contrast, patients who required grafts and those who underwent amputation had ΔT values of 3.28 ± 0.68 °C and 7.71 ± 1.89 °C, respectively. According to these findings, IRT had a prediction accuracy of 85.35% [69]. 

In the case of veterinary medicine, currently, skin grafting is performed in small animals, particularly when a large defect on a distal limb is present [68]. In murine animal models, thermography was used to evaluate the usefulness of therapeutic ultrasound on skin grafts. In the treated group, the authors found higher temperatures between 35 and 36 °C, while the control group had lower values between 33.3 and 34.3 °C. This could be interpreted as an improved tissue repair using ultrasound [71]. In another study in mongrel dogs, Lekas et al. [72] monitored coronary artery bypass grafting and found that thermal imaging can accurately identify ischemic processes due to stenosis or occlusion of the coronary artery. In this sense, ligation of the artery caused a rapid decrease in the epicardial surface temperature from 31 °C to 26–28 °C. Since the main cause of grafting failure, particularly in small animals [73], is the lack of adequate blood flow and the consequent ischemia and autophagy of the surrounding tissue, early detection of these processes using noninvasive equipment such as IRT is of utmost importance [20].

Contrary to skin grafting, flaps are skin transplants with an integral blood supply [67]. These types of surgeries have been monitored with thermal imaging to identify the so-called “hot spots” where correct vascularization is present, reaching a prediction accuracy of 100% of flap survival and ischemia identification [74]. Tools such as dynamic infrared thermography (DIRT) were used to monitor real-time skin perfusion of a pedicled thoracodorsal artery perforator flap in 21 patients in a Sjøberg et al. [75] study. Preoperative mapping of perforator vessels and evaluation of postsurgical perfusion showed that hot spots detected with DIRT corresponded to arterial Doppler sounds, while the disappearance of hot spots was associated with less audible or absent arterial sounds [75]. 

Another researcher who used DIRT was Miland et al. [76] to visualize skin perfusion in female patients undergoing abdominal skin flaps. DIRT correlated with angiography techniques to identify perfused areas of the flap. Likewise, in free flaps performed in breast reconstruction surgeries, surgeons have been using IRT, together with Doppler ultrasound and computed tomography angiography techniques, to monitor the viability of the flap but also as a novel tool to preoperatively locate perforator vessels in flaps [23]. In contrast, some studies reported that IRT was unable to detect a case of poorly perfused flap until the skin transplant was dead; nonetheless, the accuracy of the technique was estimated at 96.43% [77].

In the case of nonhuman animals, Miland et al. [78] performed skin flaps in Wistar rats and used DIRT and angiography to predict flap survival. The percentage of flap survival using both techniques was similar (63–74%), with an accuracy of 0.86–0.90%. The study also showed higher temperatures in the axial center of the flaps (around 33–34 °C) and a significant decrease in the distal ends (the lowest temperature was 30 °C), showing that the healing process starts at the edges of the flap, where vascular supply can be easily compromised. Tenorio et al. [79] used IRT in rats to recognize perfusion failure in free flaps. The authors performed ligation of one artery or vein to observe the thermal changes. The results showed that ligation of the blood vessel made the hot spot disappear, meaning a 100% chance of identifying permeable and occluded vessels. In the same way, in rats undergoing free flaps, localization of perforators in the abdominal skin was performed with IRT by identifying hot spots. Then, 24 h after flap placement, the correct blood flow through perforators was assessed with IRT and macroscopical (necropsy) observations. Necrosis occurred in places where IRT did not detect perforators in 12 of 31 cases [80].

Apart from determining the degree of damage and correct blood flow, IRT has also been used to assess the effect of several treatments on skin transplant success. This was reported in a study performed on rats receiving five different treatments (saline, capsaicin, methylprednisolone, mitomycin, and pentadecapeptide) on dorsal skin flaps [81]. Through IRT, it was found that methylprednisolone and pentadecapeptide reduced ischemic necrosis. These results were comparable to the Shejbal et al. [82] study, in which a full-thickness dorsal murine skin flap was treated with saline, capsaicin, and methylprednisolone to assess their effect on tissue vitality and survival area. Methylprednisolone resulted in a uniform temperature distribution of the skin associated with reduced flap necrosis by 56%. 

Assessing the degree of damage of a tissular injury implicates not only cell death but also damage to the surrounding blood vessels [83]. Knowing that vascular trauma affects tissular viability and also alters local heat exchange is the basis of understanding why IRT is used to provide an early evaluation of burn wounds. IRT can help surgeons to identify the extension of the damage and the most suitable treatment and to monitor surgical procedures such as skin grafting and free flaps, as summarized in Table 2. Moreover, during the postoperative period, the healing process can also be monitored with IRT to improve recovery time and propose complementary treatments and evaluation devices. Nonetheless, this has not been extensively researched in veterinary medicine. 

## 6. Infrared Thermography Used to Monitor Healing and Surgical Wound Care

While repair refers to a process where substantial tissular damage is present and the normal architecture of the cells cannot be completely restored, healing refers to the proliferation process when cells can regenerate. The healing process after surgery is another field where IRT has been used. Healing implicates several phases such as the inflammatory phase (2–5 days), proliferative phase (5 days to 3 weeks), and remodeling phase (3 weeks to years) [54]. In the case of burn injuries and skin transplants, one of the primary things to ensure transplant success is to promote correct blood perfusion [54]. Authors such as Ramírez-GarciaLuna et al. [15] mention that regarding wound care, comparisons between the surface temperature of the wound bed and the proximate healthy tissue are useful because alterations in blood flow due to inflammation or obstructed blood flow might cause an increase or decrease, respectively, in skin surface temperature.

As reported by Li et al. [54], IRT can predict healing status with a sensitivity of 91.67% and a specificity of 85.71% by monitoring local blood perfusion in thoracic surgical incisions. According to the results, healed patients maintained a higher incisional temperature between 32.3 ± 1.6 °C and 33.8 ± 0.9 °C, while unhealed patients recorded hypothermic areas between 30.0 ± 1.2 °C and 32.4 ± 0.8 °C. Moreover, IRT detected hypothermic areas in the incision surface of a patient due to local infection, which could be used as a prognosis marker [54]. Similarly, higher periwound temperatures (more than 35 °C) in humans suffering from pressure ulcers were associated with a better healing process, while poor prognosis wounds had a temperature of less than 34 °C. Differences between the wound bed and periwound surface were also greater in more severe pressure wounds [85]. The hypothermia observed in these patients is known to delay recovery time [86]. Therefore, promptly identifying the surface decrease in temperature might be helpful to intervene and reduce postsurgical complications. 

In the case of veterinary medicine, in a red wolf sustaining an injury in the left thoracic limb, Hurley-Sanders et al. [84] performed a free skin graft and evaluated through IRT the correct vascularization of the graft. The authors evaluated 2 days before surgery, immediately after surgery, and every 2–3 days within 14 postsurgical days. The results showed that graft adherence and healing were obtained when neovascularization caused a similar temperature between the graft site and surrounding healthy tissue [84]. Similarly, in Nigerian indigenous dogs, IRT was used in animals undergoing castration, otectomy, and gastrotomy [87]. The authors evaluated wound surface temperature and found that the initial temperature of castrated dogs was 33.34 ± 0.86 °C, a temperature that increased to 35.64 ± 0.57 °C at 6 h postsurgery, 35.14 ± 0.38 at 24 h, 35.82 ± 0.61 °C at 48 h, 35.24 ± 0.64 at 1 week, and progressively decreased in the second week postsurgery (32.84 ± 1.27 °C). In contrast, dogs undergoing otectomy had an initial temperature of 33.78 ± 0.86 °C that increased to 35.34 ± 1.13 °C, 34.58 ± 0.49 °C, 36.94 ± 0.31 °C, 36.58 ± 0.29, and 34.66 ± 0.74 °C at 6 h, 24 h, 48 h, 1 week, and the second week, respectively. A similar thermal response was observed in gastrotomy-subjected animals, in whom the initial value of 31.18 ± 0.86 °C progressively increased to 32.70 ± 1.02 °C, 34.10 ± 0.55 °C, 35.36 ± 0.33 °C, and 36.00 ± 1.37 °C in the same period. The higher temperatures recorded in these animals might be attributed to a higher pain and inflammatory response, as this process alters body temperature and is also a stressor that will modify peripheral blood flow [42].

Healing monitoring was also performed in piglets after CO_2_ surgical laser for castration. Viscardi et al. [88] used IRT in these Yorkshire x Landrace piglets to evaluate if surgical laser helps to reduce pain and inflammation and improve the healing process. Laser-castrated piglets had lower temperatures at the incision site than scalpel-castrated animals (*p* = 0.0005). Due to the laser, thermal lesion was observed in the piglets, along with pain-related behaviors, which would make this technique not a refinement technique for the conventional techniques. Agents to promote healing (e.g., germicide, aluminum powder, liquid bandage) have also been evaluated with IRT, as shown by Marti et al. [89] in Angus bulls undergoing castration. The scrotal surface temperature was not significantly different among groups during the 49 days of evaluation (average values between 27.5 °C and 28.5 °C), and the time for incision healing did not differ. These results might be associated with a similar inflammatory and painful response in all bulls, showing that IRT can be an adjuvant to monitor the healing period and manage pain, an important sign that also must be considered [90]. In longer-lasting pain procedures such as rubber ring castration, usually performed in domestic animals, Nogues et al. [91] evaluated wound healing and inflammation in dairy calves after surgical or rubber ring castration. Rubber-ring-castrated animals did not fully heal within the eight evaluation weeks and, according to IRT, the thermal surface response around the lesion was 1.7 ± 0.35 °C higher than the surgical group. In contrast, surgically castrated calves fully healed at week four.

These studies, apart from showing that IRT can help to determine a sustained inflammatory response associated with healing delay, show that it is a tool that can help to refine current procedures not only because they are painful but because tissue damage might be greater with a greater inflammatory response. For example, analgesic options such as lidocaine in surgical incisions were evaluated through IRT to assess if the local anesthetic has a negative effect on wound healing, as studied in cats and dogs by Herlofson et al. [87]. Surface temperatures between lidocaine alone or NaCl did not differ (*p* = 0.554) with healing scores of 3 and 2.5, respectively, showing that lidocaine addition does not influence wound healing. Therefore, the effect that surgical stress, inflammation, and pain have on local tissue perfusion and heat production is relevant and is a field that can benefit from the application of IRT. 

## 7. Technical Factors That Can Influence Thermal Imaging in Animals

It is important to consider some technical and physiological aspects that can influence the surface thermal response in animals. An example of this is the image resolution used in different studies, which might affect the quality of the data. In horse and cattle, resolutions of 80 × 90 pixels to 320 × 240 pixels were proposed [92,93,94]. In human medicine, Schuster et al. [95] reported that high-resolution thermography (320 × 340 pixels) helps to assess clinical examination in tungiasis-associated morbidity. This is due to the minimal changes in surface temperature that can be detected with high-resolution cameras. Thus, the technical properties of thermal cameras must be addressed when suggesting IRT to assess vascular disorders.

On the other hand, body emissivity is another element that is highly relevant when using IRT, since this parameter describes the ability of a body surface to emit radiation [25,96]. Several studies reported differences in skin emissivity according to the species. For example, in humans, skin emissivity is 0.98, a value that is close to pig skin (between 0.92 and 0.93) [97]. In horses and rabbits, values of 0.95 and 0.97, respectively, were mentioned [98,99]. These differences are related to the skin characteristics (e.g., presence of hair, feathers, glabrous skin) even within the same species, as discussed by Soerensen et al. [97] in pigs, in whom different emissivity values were reported in hairy skin areas (0.93), in dorsal areas (0.95), or in dead animals (0.8). In horses, Jørgensen et al. [100] mention that hair acts as a thermal insulator, which might influence heat exchange with the environment, recording lower surface temperatures. 

The presence or absence of hair and its characteristics (e.g., color, length, type, and distribution) need to be considered when using thermal imaging to assess peripheral blood flow disorders in domestic and nondomestic animals [101]. For example, Kwon and Brundage [102] compared the surface temperature of dogs with different types of hair coats: short, curly, long, and double-coat types. The authors found that animals with short hair had the highest surface temperatures (31.77 ± 0.19 °C) and that these values were approximately 2 °C above the temperatures of the other animals. This could be related to short hair not providing a thicker insulative layer, in contrast to long or double-coat animals, which would permit and make more easily recognizable an increase in IRT values. Nonetheless, another study performed on pregnant and nonpregnant mares with different hair-coat features did not find significant differences regarding hair length [103]. Furthermore, as mentioned by Favilla and Costa [104], thermoregulatory strategies also differ among species. In the case of marine mammals, heat dissipation is performed through the peripheral vascularization of the flippers and flukes. In contrast, birds exposed to heat stress tend to dissipate heat through the bill (beak) [105,106]. Moreover, Gauchte et al. [105] mention that it is not only the species or the presence of hair/feathers that influences the thermoregulatory response of animals but also external factors such as wind speed, ambient temperature, sun exposure, and shooting distance that can affect infrared readings.

To address the influence that hair presence and type have on IRT values, the use of certain thermal windows has been proposed. These regions are characterized by a rich network of superficial capillaries and arteriovenous anastomosis that facilitate heat exchange and also tend to be zones without hair [25]. The ocular surface temperature is one of the most-used thermal windows to evaluate the health state, stress process, and pain perception in several species [107,108,109]. Nonetheless, the evaluation of a peripheral ischemic process and its influence and other thermal windows that are not close to the ischemic zone has not been reported in animals.

The consideration of environmental elements that can influence thermal imagining is another issue that needs to be addressed. For example, in dairy cattle, it was reported that a wind speed of 7 km/h or 12 km/h can decrease the ocular surface temperature by up to 0.43 °C and 0.78 °C, respectively. Likewise, direct solar radiation can increase the surface temperature of animals by 0.56 °C [110,111]. To reduce the effect of these elements, signal-processing techniques are proposed to improve the reliability of IRT. The use of cameras that control the automatic brightness could provide clearer pictures when thermal imaging is performed under field conditions (e.g., in farm animals where environmental factors cannot always be controlled) [112]. Other processing techniques such as wavelet transforms and signal reconstruction, among others, are currently applied in industrial thermal imaging to improve IRT reliability [113]. Nonetheless, the application of these techniques in veterinary medicine is still needed to improve its use in animals. 

## 8. Perspectives on the Use of IRT in Veterinary Medicine

The discussion presented shows that IRT is sensitive to detecting changes in both global and local perfusion. While it has been reported as a tool to recognize hypoperfusion states during pathological thromboembolism or diabetic foot, it is necessary to consider whether it is possible to apply this tool as a reliable and sensitive diagnostic method focused on any of these pathologies [14]. Nonetheless, since its application in veterinary medicine to monitor vascular diseases is limited, further research could aim to evaluate patients with lupus or vasculitis. Moreover, necrotic tissue monitoring observed in patients attacked by venomous animals such as snakes or spiders could be another field for investigation. 

Because these peripheral circulatory alterations can affect extremities with vascular compromise, IRT could be implemented as a method to decide whether a limb needs amputation or not [44]. In veterinary medicine, this field of research is limited, and additional studies would be needed to validate the use of IRT for wound care and treatment decisions in animals. Moreover, a field where IRT has not been applied yet in veterinary medicine is the viability of organ transplant surgeries, where monitoring adequate blood perfusion could be related to the success of the surgical procedure.

Although IRT has been used in human medicine to evaluate orthopedic conditions and postoperative outcomes related to joint reconstructions or implant placements [70], in veterinary medicine, only a couple of studies have been published. Examples of these are Zhou et al.’s [114] preclinical study on dogs undergoing bone autografts, in whom an infrared camera was used to find the speed of drilling that resulted in less thermal-mechanical damage to the bone cell viability and proliferation. The authors found that local increases in the cortical surface temperature of 2–6 °C with 500 rpm drilling were associated with enhanced cell performance and osteogenic gene expression, showing that thermal evaluation of these procedures might help to improve the quality of bone autografts. Similarly, the cortical bone surface temperature of sheep receiving implants placed in the iliac crest was evaluated to determine the effect of different osteotomy drilling speed protocols. It was found that although 50 rpm drilling had the highest bone temperature and the highest cell stability, all drilling protocols showed implant stability regardless of the temperature changes [115]. Furthermore, since some studies report that bone implants can increase the local surface temperature within the first week after the surgical procedure as an indicator of the repairing phase, IRT could be applied in animal patients to monitor orthopedic interventions [116]. 

Likewise, it is necessary to consider the limitations of the technique. The administration of drugs such as local anesthetics can alter the thermal response and could be misinterpreted as a pathological effect instead of a drug-related response. Furthermore, IRT indirectly assesses the amount of radiated heat, and this depends on external elements such as environmental temperature, and wetness of the surface, among others [117]. Therefore, IRT is recommended as a complementary tool that should be used with other techniques (e.g., angiography, ultrasound, or histopathologic analysis), since some studies have mentioned that IRT does not have a higher sensitivity than traditional imaging techniques such as radiography, ultrasonography, or magnetic resonance in neurological patients [118]. For example, Han et al. [119] concluded that indocyanine green angiography performed in a rat perforator flap model predicts tissue necrosis with an accuracy of 92%, in contrast to thermal imaging (74%) and near-infrared spectroscopy (71%). Thus, a comprehensive evaluation of peripheral vascular alterations and tissue viability in animal medicine requires additional technologies. 

## 9. Conclusions

IRT is a technique that can noninvasively and indirectly detect peripheral vascular disorders and the consequent alterations to the amount of radiated heat from the skin. Although the application of IRT to study vascular integrity and tissue viability is more frequent in human medicine, the current research performed in animals has shown that IRT can differentiate healthy from sick patients in cases of thrombosis, thromboembolisms, and ischemia processes.

Additionally, the microvascular alterations that can be found as a result of burn wounds is a field where IRT can help in determining the degree of damage and predict healing success in cases where conservative treatment or skin transplant is optional. In this sense, higher differences between wound bed surface temperature and healthy tissue have been associated with a higher degree of tissular damage. Likewise, a hypothermic local response is related to delayed healing and possible postsurgical complications such as incisional infection. 

Since the lack of an integrated network of blood perfusion affects tissue viability and the wound healing process, IRT can be suggested as a complementary tool to other techniques such as angiography or Doppler ultrasound to assess vascular integrity or to promptly adopt treatment options.

## Figures and Tables

**Table 1:** Technical and experimental characteristics of animal studies using IRT to evaluate vascular disorders.

Species	Camera	Distance (cm)	Emissivity	Ambient Temperature (°C)	Aim	Outcome
Cats [14]	Flir C2	75	0.95	20.0	Identify aortic thrombosis.	2.4 °C to differentiate sick animals.
Dogs [52]	FlirOne Pro	30	NR	25.0	Diagnose thromboembolism.	Affected limb had lower temperature (31.3 °C vs. 35.0 °C).
Pigs [49]	FlirOne	NR	NR	NR	Establish coronary circulation.	Visible occlusion of coronaries appearing in bright yellow.
Pigs [45]	Flir-E6	50	0.98	NR	Assess changes in skin temperature in response to blood pressure.	Negative correlation between blood pressure and temperature gradient.
Rats [50]	Flir T650SC	45	NR	NR	Monitor pedicled island perforator flaps.	1.4% increase in red zones or hot spots.
Rats [51]	Flir 335	20	NR	NR	Detect tissue perfusion disorders in femoral vessels.	Return of warmth in the limb was slower.

NR: not reported.

**Table 2:** Technical and experimental characteristics of animal studies using IRT to assess burn depth and skin transplant survival.

Species	Camera	Distance (cm)	Emissivity	Ambient Temperature (°C)	Aim	Outcome
Pigs [59]	Flir T300	30	NR	NR	Assess burn depth.	Surface hypothermia (27.31 ± 0.37 °C) predicts larger scar depth.
Pigs [60]	ADT	NR	NR	NR	Assess burn depth.	Shorter thermal time (49.1 ± 23 s) related to fast healing.
Pigs [65]	Flir A655sc	40	NR	NR	Assess burn severity.	Severe burns have lower temperatures (32.4–33 °C).
Rats [76]	DIRT Flir ThermaCAM S65	NR	NR	NR	Predict flap survival.	74% flap survival.
Rats [79]	BioScanIR	NR	NR	NR	Detect flap failure.	Hot spot disappeared, followed by macroscopic congestion.
Red wolf [84]	eVs DTIS 500	NR	NR	NR	Monitor free skin graft.	Neovascularization in the graft becomes thermally equivalent to healthy tissue.

ADT: active dynamic thermography; DIRT: dynamic infrared thermography; NR: not reported.

## Data Availability

Not applicable.
